# A Global Network Meta-Analysis of the Promotion of Crop Growth, Yield, and Quality by Bioeffectors

**DOI:** 10.3389/fpls.2022.816438

**Published:** 2022-03-01

**Authors:** Michelle Natalie Herrmann, Yuan Wang, Jens Hartung, Tobias Hartmann, Wei Zhang, Peteh Mehdi Nkebiwe, Xinping Chen, Torsten Müller, Huaiyu Yang

**Affiliations:** ^1^College of Resources and Environment, Academy of Agricultural Sciences, Southwest University, Chongqing, China; ^2^Institute of Crop Science, University of Hohenheim, Stuttgart, Germany; ^3^Interdisciplinary Research Center for Agriculture Green Development in Yangtze River Basin, Southwest University, Chongqing, China; ^4^Crop Production, Landwirtschaftskammer des Saarlandes, Bexbach, Germany

**Keywords:** biostimulants, biofertilizers, sustainable agriculture, nutrient use efficiency, pH, PGPR

## Abstract

Bioeffector (BE) application is emerging as a strategy for achieving sustainable agricultural practices worldwide. However, the effect of BE on crop growth and quality is still controversial and there is still no adequate impact assessment that determines factors on the efficiency of BE application. Therefore, we carried out a network metaanalysis on the effect of BEs using 1,791 global observations from 186 studies to summarize influencing factors and the impact of BEs on crop growth, quality, and nutrient contents. The results show that BEs did not only improve plant growth by around 25% and yield by 30%, but also enhanced crop quality, e.g., protein (55% increase) and soluble solids content (75% increase) as well as aboveground nitrogen (N) and phosphate (P) content by 28 and 40%, respectively. The comparisons among BE types demonstrated that especially non-microbial products, such as extracts and humic/amino acids, have the potential to increase biomass growth by 40–60% and aboveground P content by 54–110%. The soil pH strongly influenced the efficiency of the applied BE with the highest effects in acidic soils. Our results showed that BEs are most suitable for promoting the quality of legumes and increasing the yield of fruits, herbs, and legumes. We illustrate that it is crucial to optimize the application of BEs with respect to the right application time and technique (e.g., placement, foliar). Our results provide an important basis for future research on the mechanisms underlying crop improvement by the application of BEs and on the development of new BE products.

## Introduction

The use of bioeffectors (BEs) has been proposed as a promising solution for the challenges of sustainable agriculture (Povero et al., 2016). A BE is defined as organic material and/or microorganisms applied to living plants or soil to enhance nutrient uptake, stimulate growth, improve stress tolerance and crop-quality traits, regardless of its nutrient contents (Van Oosten et al., 2017; Rouphael and Colla, 2018; Schütz et al., 2018; Ricci et al., 2019). Typical BEs include amino acids and other organic compounds, seaweed extracts, and botanicals as well as microorganisms including fungi and bacteria. Numerous studies have demonstrated that the application of BEs is beneficial to plants in all developmental stages (Qiu et al., 2020; Rouphael and Colla, 2020b) and may increase yield (Schütz et al., 2018).

Microbial products can directly influence nutrient availability in soil or nutrient uptake by plants. For example, N-fixing bacteria are able to sequester nitrogen (N_2_) from the air, thereby increasing the pool of reactive N in the soil. Phosphate (P)-solubilizing bacteria (PSB) can transform phosphate from insoluble compounds to bioavailable ones by releasing protons, exudates, such as chelates, and other substances. Previous studies have determined that symbiotic arbuscular mycorrhiza fungi (AMF) promoted phosphate, nitrogen, and micronutrient acquisition through spatial enlargement of the influence of the rhizosphere (Watts-Williams and Cavagnaro, 2014). The stimulation of plant nutrient acquisition by BEs is beneficial, especially under nutrient-poor conditions and it reduces the dependency of agriculture on the application of inorganic fertilizers (Oldroyd and Leyser, 2020). Further, in high-input agricultural systems, the application of BEs can facilitate fertilizer and pesticide use without risking yield decline. Some plant growth-promoting microbes can alter the hormone balance in plants and improve plant performance. Meents et al. (2019), for instance, reported that the beneficial fungi *Piriformospora indica* increased auxin levels in plants thereby promoting root growth.

Besides selected microbial organisms, BEs also comprise a variety of processed substances from soil and plants, such as humic acids, amino acids, and extracts, from seaweeds and other plants. These products are diverse in terms of their potential influence on plants and the microbial community in the soil. Extracts can directly influence plant growth by bioactive compounds or indirectly by ameliorating soil characteristics (Shukla et al., 2018). By altering soil water holding capacity, they indirectly improve soil growing conditions and nutrient availability for plants (Khan et al., 2009). Extracts also interact with the soil microbial community and promote the function of beneficial fungi (e.g., AMF) by increasing infectivity, spore production, colonization, and hyphae growth (Khan et al., 2009). Humic and amino acids can stimulate the proton pump of the plant (Jindo et al., 2012), thereby supporting the plant’s own nutrient solubilizing pathway and increasing nutrient availability in the rhizosphere. Additionally, the complexation of micronutrients by organic acids, reduction of Fe(III) to Fe(II), and the presence of organic radicals and phenolic compounds are further possible explanations for an increased nutrient availability in soil after the application of organic acids (Adani et al., 1998).

BEs have further been shown to improve plant tolerance to abiotic and biotic stress. Two main pathways are described: Firstly, beneficial microbes can adapt themselves to extreme conditions, such as salinity and cold temperatures (Selvakumar et al., 2008; Upadhyay et al., 2009). Microbes can then increase the tolerance of plants to abiotic stress by ameliorating plant performance under these conditions (Van Oosten et al., 2017). Secondly, microbes and natural compounds can produce substances that promote stress adaptation by the plant (Van Oosten et al., 2017).

Considering the recent public awareness on environmental degradation and pollution by agricultural production, the topic of BE-use in sustainable agriculture is becoming increasingly important. However, BEs as living organisms or natural extracts are influenced by edaphic and environmental factors after application. This leads to unreliable outcomes for farmers, thereby limiting their practical use and adoption. Despite decades of research on this topic, the efficiency of BEs varies widely among published studies (Schmidt and Gaudin, 2018). In particular, the pivotal factors determining the effects of BEs, such as crop specificity and mode of action, are still unclear. Moreover, most data from Chinese BE studies are not included in previously published metaanalyses.

The goal of this study was to systematically quantify the improvement of plant performance and other important indicators (e.g., quality, nutrient acquisition) *via* BE application in general, but also under given local agricultural conditions (e.g., pH, crop type). Another aim was to specify practical and efficient application strategies of BEs. To achieve these goals, a network metaanalysis was conducted to primarily determine whether BEs show a global positive effect on plant biomass, yield, aboveground nutrient content, nutrient use efficiencies, and quality indicators (protein- and soluble-solids content). Secondly, crucial factors that influence the efficiency of BEs were determined. Therefore, we analyzed variables, such as crop type, edaphic pH, and other aspects, in crop production on a global scale.

## Materials and Methods

### Data Collection

In this metaanalysis, we compiled a global dataset by retrieving peer-reviewed studies published until December 2021 using Scopus by Elsevier, ISI-Web of Science, the search engines of Microsoft Academic, and Hohsearch (search engine of the University of Hohenheim, Stuttgart, Germany). Studies in Chinese were extracted from the China Knowledge Resource Integrated Database (CNKI). “Biofertilizer,” “biostimulant,” or “bioeffector” together with “nutrient” or “quality” were selected as keywords. This resulted in more than 2,000 possibly fitting studies. Studies were sorted by relevance prior to checking them. Literature searches were terminated if 20 studies in succession did not fit into the inclusion criteria defined below. We filtered the studies by the following inclusion criteria: (i) the standard errors and the number of replicates that were reported. These values were needed to calculate the effect sizes. (ii) The experiment had to be laid out in the soil. (iii) The only difference between the control and the treatment group was the BE application. (iv) Germination trials were excluded. (v) The study stated at minimum two predefined response variables. (vi) The agricultural crops were used for food, fodder, or biomass production. A PRISMA flowchart is included in the Supplementary Materials (Supplementary Figure 1).

### Preprocessing of Data

When data were available in graphs only, the program GetData Graph Digitizer (Ver. 2.22) was used to extract means and standard errors. To convert the concentration of available N and P in soil from g kg^–1^ to kg ha^–1^, the bulk density was estimated according to the United States Department of Agriculture – Natural Resources Conservation Service (2019). The effective root zone was determined as 0.5 m. If only the total N of the soil was analyzed, then the available N was estimated based on the calculation by Sponagel et al. (2005). When organic fertilizers were used and in case their nutrient composition was not analyzed, estimated nutrient contents were used from Herbert et al. (2019). If it could not be estimated, the study was not considered in the investigations of nutrient-use efficiency. Additionally, the shoot biomass for the calculation of nutrient use efficiencies was transformed into kg ha^–1^ by considering plant densities indicated in the papers or estimated based on a common agricultural practice (Supplementary Table 1). pH values were adapted in case it was measured in CaCl_2_ or KCl, according to Reuter et al. (2008), to convert values to measurements in water. If the method was not given, it was assumed to be measured in water. For all the other measurements, methods were considered similar, and no transformation of the values was implemented.

The potential influencing variables BE, geographic region, application strategy, and application time were categorized. The BEs including microorganisms were categorized according to Schütz et al. (2018). Botanicals, as well as sea-weed extracts, were summarized as the group of “extracts.” Humic/amino acids formed another category. Substances not fitting into these categories were pooled in the group “others.” The combination treatment was subdivided into a dual combination, which included two different microorganism categories, a combination of more than two microorganism categories, and a mixture of microorganisms and non-microbes.

In the case of BE application, it is very likely that different effects must be expected for different management regimes. As nutrient balances vary largely across the world and locally adapted strategies are needed (Haygarth and Rufino, 2021), the World Bank (2019) classification of regions was applied to account for this variation. This categorization of the countries was chosen as a broad indicator for the agricultural practice in the countries. According to the set of the experiment, we divided the data into pot and field trials. The grouping into perennial or annual did not happen according to the biological capability of growing several seasons, but according to the layout of the experiments. If the plants in the experiment had been growing for several seasons or the BE application was conducted for more than 1 year on the same plant, the study was identified as perennial.

Furthermore, the data were subdivided according to the application strategy. We identified four different modes of application. The BEs were applied either (1) to the seed, (2) in the soil, (3) to the seedling, or (4) by foliar application. Additionally, a combination of two or more above-mentioned modes of application was also considered. Concerning application time, we distinguished between a single application, before/at sowing or after sowing, or a multiple application. The pH range was divided into five subgroups, namely, ≤ 5.5 “strongly acidic,” 5.5 < pH ≤ 6.5 “slightly acidic,” 6.5 < pH ≤ 7.5 “neutral,” 7.5 < pH ≤ 8.5 “slightly alkaline,” pH > 8.5 “strongly alkaline.”

### Statistical Approach

Determination of effect sizes is one option in metaanalyses to handle the variation of units and measurements and to enable the calculation of overall effects, thereby enabling the comparison of effects between studies (Sánchez-Meca and Marín-Martínez, 1998). For calculating the effect size, the treatment means were standardized by dividing them by their standard error. Additionally, the correction of bias according to Hedges (1981), which includes the repetition number, was added to the calculation. A logarithmic transformation was then applied to further stabilize the variance and counteract the right-skewness of the effect sizes. Effect sizes in the current study were calculated for the following traits: yield, shoot biomass, root biomass, aboveground nutrient content of N and P, nutrient use efficiency of N and P, as well as the quality parameters of protein and soluble solids content. We defined shoot biomass as the total aboveground plant material and yield as the main marketable product. Nutrient use efficiency was defined as the biomass divided through the total amount of available N and P in soil, respectively (available nutrient in soil + input through fertilizers).

**FIGURE 1:**
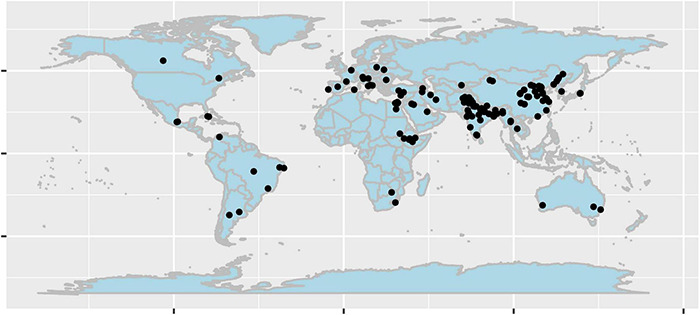
Location of studies included in the data set. The figure was created by the R package ggmap (Kahle and Wickham, 2013).

Publication bias can strongly affect the results of metaanalyses, thereby altering or overestimating the outcomes (Levine et al., 2009). It was assumed that articles will be more likely to be published if they prove positive effects, similar to medical studies (Joober et al., 2012). In our study, when a strong right skewness was observed, the statistical approach by Carrijo et al. (2017) was followed. Therefore, independently for every group of effect size, when the effect sizes of studies exceeded the mean plus three times the standard error, they were excluded from the analysis. After the exclusion, 186 out of 197 studies were used for the final analysis including a total amount of 1,791 observations. For the first time, 64 studies conducted in China (423 observations) were included. The locations of those studies when identifiable are shown in Figure 1. The 186 studies, which were finally included in the metaanalysis, are listed in the references.

It is important to consider the methodological quality of the studies as it could strongly influence the outcome of a metaanalysis (Moher et al., 1998). Therefore, we included the impact-factor of the journal, where the study was published as a covariate in our model. In later steps, the covariate was again removed from the model, as it was not significant in any case.

In the collected data structure, several treatments correspond to the same control. Therefore, to quantify the impact of each BE type of one study and still avoid correlation of effect sizes, a network metaanalysis was necessary using a linear mixed model described by Madden et al. (2016). This approach has the advantage that it enables us to compare treatments indirectly even if they never occurred together in the same study. A random study effect was included in the model as just a sample of available studies was chosen, and the aim of the analysis was to generalize our result for future studies to be published. The model can be described as follows:


$${\text{y}}_{\text{ijklmnopqr}}=\mu +b_{\text{j}}+\tau_{\text{i}}+{\left(\alpha \tau \right)}_{\text{ik}}+\beta_{\text{li}}+\gamma_{\text{mi}}+{\left(\delta \tau \right)}_{\text{in}}+\theta_{\text{oi}}+{\left(\vartheta \tau \right)}_{\text{iq}}+{\left(\phi \tau \right)}_{\text{iq}}+{\left(\kappa \tau \right)}_{\text{ir}}+{\text{e}}_{\text{ijklmnopqr}},$$

where μ is the intercept, b_j_ is the random effect of the jth study, τ_i_ is the main effect for the ith treatment (with levels control and treatment). β_li_, γ_mi_, and θ_oi_ are the effect of the lth application time, the mth BE, and the oth mode of application, respectively. These factors are nested within treatment, as in the control BEs were not applied at all. (ατ)_ik_, (δτ)_in_, (ϑτ)_iq_, (ϕτ)_iq_, and (κτ)_ir_ are the interaction effects of the ith treatment and kth testing system, the nth crop, pth perennial or annual, qth geographical region, and the rth pH-effect, respectively. We did not fit the main effects for these latter terms as we were only interested in the differences of control and treatment depending on these factors. The value e_ijklmnopqr_ is the error of mean effect size y_ijklmnopqr_ with a homogeneous error variance.

For all factors in the model above, our main focus was on the differences between control and treatment, and differences between these differences across treatments. Contrasts were used to calculate the difference of the differences between control and treatment. As the data were logarithmically transformed prior to analysis, differences correspond to ratios on the original scale. The back-transformed value from a difference of differences corresponds to an odds ratio.

To avoid overfitting, which means that additional non-significant factors in the model mask the effects of other factors, we performed a model selection. To implement this, we switched the method for variance component estimation to maximum likelihood. We then used the Akaike Information Criterion (AIC, Wolfinger, 1993) selection criterion to find the best model for our data. Since the AIC can only be used for comparisons of models which have identical data sets, an auxiliary dataset was created by omitting all observations for which data for at least one of the parameters of the full model was lacking. Afterward, the model selection was carried out using this auxiliary dataset. It was assumed that the best-selected model through the model selection is transmissible to the entire dataset later since studies were considered as random samples.

In none of the final models from the model selection, BE types were included as factors. Since one of our initial focuses was to investigate the differences between BE types, selected models of shoot and root biomass, yield, aboveground P as well as N content, and nutrient use efficiencies were expanded by the BE type. This resulted in slightly higher AIC and changed mean effects (Supplementary Table 2). Further analyses for all other factors were conducted with the non-expanded model and only BE types were investigated using the expanded model.

After finding the best model *via* AIC, the restricted maximum likelihood estimation was applied (Patterson and Thompson, 1971). Confidence limits were calculated for each difference between treatment and control and the contrasts of the differences among treatments. We considered estimates to be significantly different from zero when their p-Values were below or equal to 0.05, and in tendency when their p-Values were below or equal to 0.1. Afterward, differences and contrasts [as well as their lower and upper bounds of the confidence limit (α = 0.05)] were backtransformed to the original scale for presentation purposes only. Standard errors were backtransformed using the approximate delta method (Xu and Long, 2005). Normal distribution and homogeneous variances of residuals (on the transformed scale) were checked graphically.

The illustration of the graphs was implemented in R with the package ggplot2 (Wickham, 2016). The figures depict the median and confidence limits of the subgroup analysis, as well as the overall effects, which we defined as the difference of the main effects τ_i_, namely the difference of treatment and control factors.

## Results

The results of the model selection are shown in the Supplementary Materials (Supplementary Table 3). In the root biomass investigation, no data was available for perennial crops and field trials. Those parameters were therefore not considered in the analysis. The country grouping was also not relevant for the model of root biomass as experiments were exclusively laid out under controlled greenhouse conditions. A summary of the significant parameters in each model and the most important influencing factors for each indicator are provided (Supplementary Table 4).

### Assessment of Bioeffector Effects on Crop Growth and Yield

**FIGURE 2:**
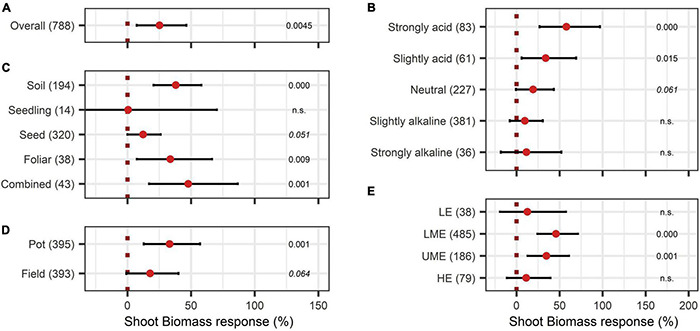
Response of shoot biomass. **(A)** Overall biomass effect of all observations (median). **(B–E)** are subgroup analyses on biomass response: **(B)** pH categories, **(C)** mode of application, **(D)** test system, **(E)** country grouping (LE: low income economies, LME: lower middle income economies, UME: upper middle income economies, HE: high income economies). Dots mark the median, lines mark the 95% confidence interval. If the line crosses the vertical dotted line, the impact of bioeffectors is not significant (α = 0.05). Numbers beside are the *p*-Values. *p*-Values > 0.1 were denoted as non-significant (n.s.), *p*-Values between 0.1 and 0.05 were shown in italics to indicate tendencies and *p*-Values ≤ 0.05 indicating significance were shown in regular font. The number of observations per treatment is indicated by the number in brackets.

As shown in Figure 2A, shoot biomass was significantly enhanced due to BE application by 26%. The variable soil pH influenced the outcome clearly (Figure 2B). A significant increase of biomass by BE application was observed in slightly acidic and strongly acidic soils, with strongly acidic soils showing the highest median of 58% increase. Regarding the mode of application, BE input *via* soil (38% increase) and foliar application (34% increase) as well as a combination of several different modes (48% increase) was more beneficial for plant biomass production than seed inoculation or application during early plant developmental stages (12% and 0.6% increase) (Figure 2C). Figure 2D illustrates that a significant increase of shoot biomass occurred in pot trials (33% increase). The efficiency of BE application differed among countries (Figure 2E), with middle-income economies obtaining the highest efficiencies (46% increase for lower-middle-income economies and 35% increase for upper-middle-income economies).

**FIGURE 3:**
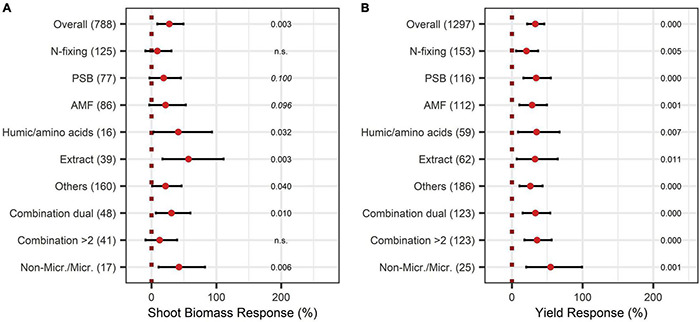
Response of shoot **(A)** and yield **(B)** for different BE types according to the expanded model. Dots mark the median and lines mark the 95% confidence interval. If the line crosses the dotted line, the impact of bioeffectors is not significant (α = 0.05). Numbers beside are the *p*-Values. *p*-Values > 0.1 were denoted as non-significant (n.s.), *p*-Values between 0.1 and 0.05 are shown in italics to indicate tendencies, and *p*-Values ≤ 0.05 indicating significance were shown in regular font. The number of observations per treatment is indicated by the number in brackets.

When adding the BE types to the model, non-microbial products attained the highest responses with a shoot biomass increase of 57%, 41%, and 42% for extracts, humic and amino acids, and the combination of microbes and non-microbial substances, respectively (Figure 3A).

**FIGURE 4:**
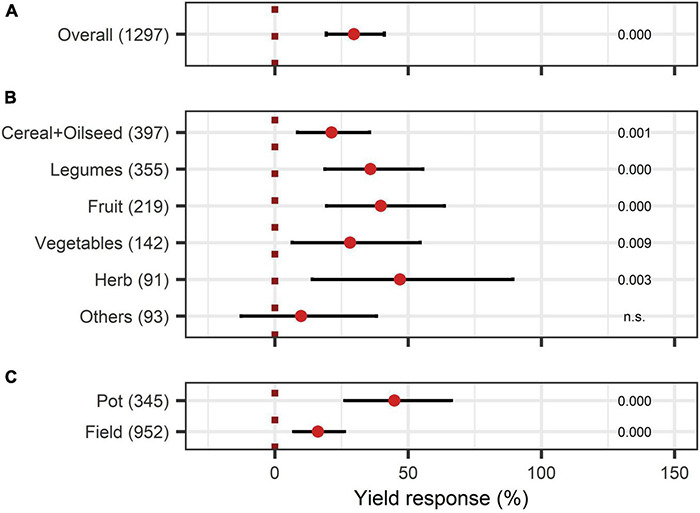
Response of yield. **(A)** Overall yield effect of all observations (median), **(B,C)** are subgroup analyses on yield response: **(B)** crop types, **(C)** test system. Dots mark the median and lines mark the 95% confidence interval. If the line crosses the dotted line, the impact of bioeffectors is not significant (α = 0.05). Numbers beside are the *p*-Values. *p*-Values > 0.1 were denoted as non-significant (n.s.), *p*-Values between 0.1 and 0.05 are shown in italics to indicate tendencies, and *p*-Values ≤ 0.05 indicating significance were shown in regular font. The number of observations per treatment is indicated by the number in brackets.

The yield was significantly augmented by 30% (Figure 4A). Almost all crop types responded significantly to BE application, except the group “others.” Herbs and fruits increased in yield the most with 47% and 40% (Figure 4B). Pot-trials obtained a significantly higher yield increase than field trials with a 45% and 16% increase, respectively (Figure 4C). When investigating the expanded model, all BE types induced significant yield increases (Figure 3B), and the combination of microbes and non-microbial products stood out with an increase of 55% (Figure 3B).

**FIGURE 5:**
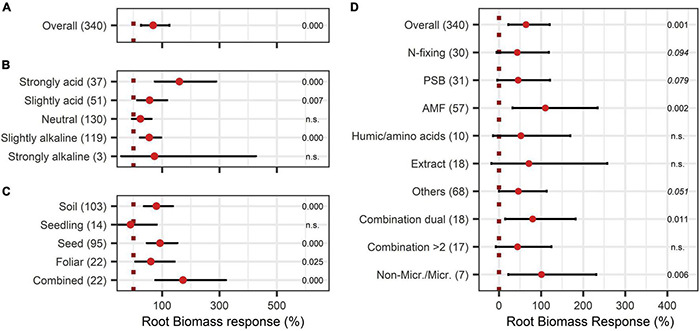
Response of Root biomass. **(A)** Overall root biomass effect of all observations (median), **(B,C)** are subgroup analyses on root biomass response: **(B)** pH categories, **(C)** mode of application. **(D)** BE types and overall median according to the expanded model. Dots mark the median and lines mark the 95% confidence interval. If the line crosses the dotted line, the impact of bioeffectors is not significant (α = 0.05). Numbers beside are the *p*-values. *p*-Values > 0.1 were denoted as non-significant (n.s.), *p*-Values between 0.1 and 0.05 are shown in italics to indicate tendencies, and *p*-Values ≤ 0.05 indicating significance were shown in regular font. The number of observations per treatment is indicated by the number in brackets.

Root biomass responded positively to BE application with an increase of 69% (Figure 5A). As shown in Figure 5B, in strongly acidic soils with pH levels < 5.5, root growth was extremely enhanced by 161%, whereas in other pH levels the effect was below the overall median. For strongly alkaline soils, data was limited in the dataset. Therefore, no definite statement can be given. The comparison of the mode of application resulted in significant increases for nearly every application mode, except the inoculation of the seedling (Figure 5C). The combination of several modes of application resulted in exceptionally high root growth with a 173% increase. The analysis of the model extended with the variable BE type led to no clear differentiation among the BE types except for AMF as well as the combination of microbes and non-microbial substances, which led to the highest root biomass improvements (Figure 5D).

### Assessment of Bioeffector Effects on Crop Quality Indicators

**FIGURE 6:**
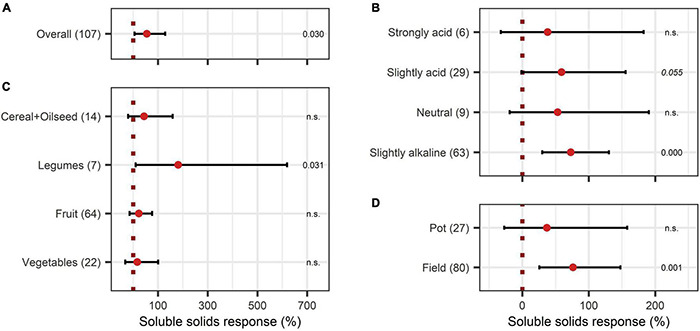
Response of soluble solids. **(A)** Overall soluble solids increase of all observations (median), **(B–D)** are subgroup analyses on soluble solids response: **(B)** pH categories, **(C)** crop type, **(D)** test system. Dots mark the median, lines mark the 95% confidence interval. If the line crosses the dotted line, the impact of bioeffectors is not significant (α = 0.05). Numbers beside are the *p*-Values. *p*-Values > 0.1 were denoted as non-significant (n.s.), *p*-Values between 0.1 and 0.05 are shown in italics to indicate tendencies, and *p*-Values ≤ 0.05 indicating significance were shown in regular font. The number of observations per treatment is indicated by the number in brackets.

**FIGURE 7:**
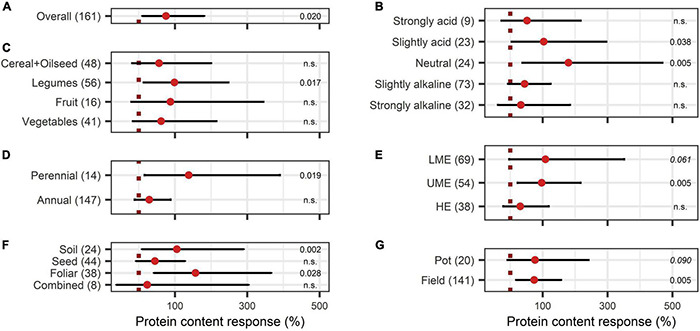
Response of protein content. **(A)** Overall protein increase of all observations (median), **(B–G)** are subgroup analyses on protein response: **(B)** pH categories, **(C)** crop types, **(D)** perennial or annual **(E)** country grouping (LME: lower-middle-income economies, UME: upper-middle-income economies, HE: high income economies), **(F)** mode of application, **(G)** test system. Dots mark the median and lines mark the 95% confidence interval. If the line crosses the dotted line, the impact of bioeffectors is not significant (α = 0.05). Numbers beside are the *P*-values. *P*-values > 0.1 were denoted as non-significant (n.s.), *p*-Values between 0.1 and 0.05 are shown in italics to indicate tendencies, and *p*-Values ≤ 0.05 indicating significance were shown in regular font. The number of observations per treatment is indicated by the number in brackets.

Concerning the crop quality improving aspects after BEs application, soluble solids content and protein content were investigated. Soluble solids showed a positive increase with an overall BE effect of 55% (Figure 6A) and also, with BE application protein content strongly increased by 75% (Figure 7A). The content of soluble solids depended on the test system, crop type, and pH (Figure 6), whereas protein content was additionally affected by the mode of BE application, the geographic region, and if the plant was perennial or annual (Figure 7). Here, foliar application resulted in a strong increase in protein content by 157%, whereas seed and soil application led to increases of 45% and 105%, respectively (Figure 7F). Crop types, such as fruits and vegetables, for which quality aspects are important for selling, showed no significant increase in soluble solids content as well as in protein content by BE application (Figures 6C, 7C). In legumes, soluble solids content was significantly enhanced by 181% and protein content by 99% (Figures 6C, 7C). Protein content was more increased in perennial crops (138%) than in annual crops (29%) (Figure 7D). From slightly acidic to slightly alkaline environments, the median responses of soluble solids content were the most pronounced with increases from 53% to 73%, but significant responses could be obtained only in slightly alkaline conditions (Figure 6B). Additionally, protein contents were significantly increased in neutral environments by 180%, whereas increases were clearly lower in other soil pH ranges (Figure 7B). The median increase of soluble solids content in the field (76%) compared to pot trials (37%) due to BE application was higher and more significant (Figure 6D), whereas for protein content, results were similar (74% for field and 77% for pot trials) (Figure 7G).

### Assessment of Bioeffector Effects on Crop Nutrient Content and Nutrient Use Efficiency

**FIGURE 8:**
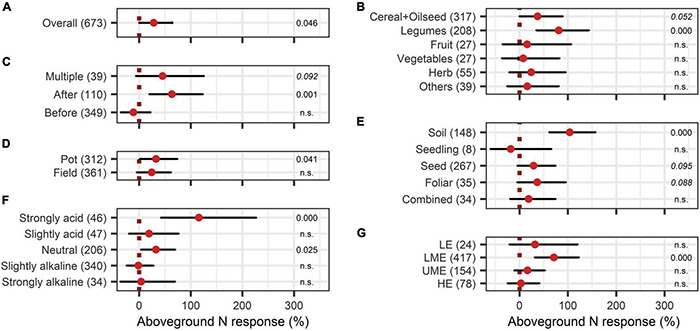
Response of aboveground N content. **(A)** Overall aboveground N increase of all observations (median), **(B–G)** are subgroup analyses on aboveground N response: **(B)** crop type, **(C)** time of application relative to sowing, **(D)** test system, **(E)** mode of application, **(F)** pH categories, **(G)** country grouping (LE: low-income economies, LME: lower-middle-income economies, UME: upper-middle-income economies, HE: high-income economies). Dots mark the median, lines mark the 95% confidence interval. If the line crosses the dotted line, the impact of bioeffectors is not significant (α = 0.05). Numbers beside are the *p*-Values. *p*-Values > 0.1 were denoted as non-significant (n.s.), *p*-Values between 0.1 and 0.05 are shown in italics to indicate tendencies and *p*-Values ≤ 0.05 indicating significance were shown in regular font. The number of observations per treatment is indicated by the number in brackets.

**FIGURE 9:**
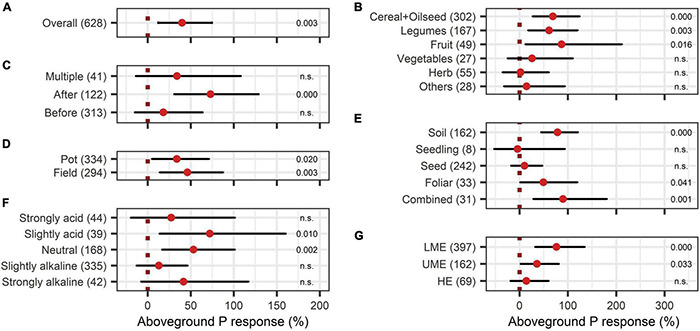
Response of aboveground P content. **(A)** Overall aboveground P increase of all observations (median), **(B–G)** are subgroup analyses on aboveground P response: **(B)** crop type, **(C)** time of application relative to sowing, **(D)** test system, **(E)** mode of application, **(F)** pH categories, **(G)** country grouping (LE: low-income economies, LME: lower-middle-income economies, UME: upper-middle-income economies, HE: high-income economies). Dots mark the median and lines mark the 95% confidence interval. If the line crosses the dotted line, the impact of bioeffectors is not significant (α = 0.05). Numbers beside are the *P*-values. *P*-values > 0.1 were denoted as non-significant (n.s.), *p*-Values between 0.1 and 0.05 are shown in italics to indicate tendencies, and *p*-Values ≤ 0.05 indicating significance were shown in regular font. The number of observations per treatment is indicated by the number in brackets.

**FIGURE 10:**
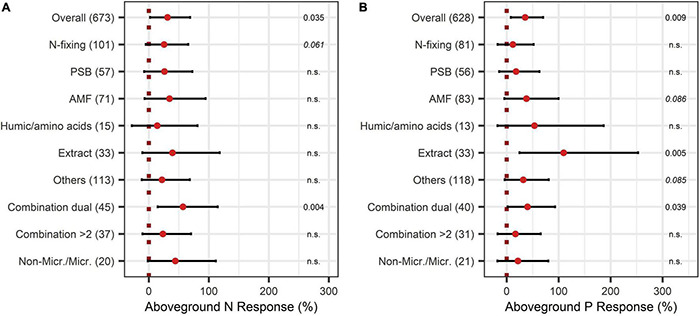
Response of aboveground N **(A)** and P **(B)** content by different BE types. Dots mark the median, lines mark the 95% confidence interval. If the line crosses the dotted line, the impact of bioeffectors is not significant (α = 0.05). Numbers beside are the *P*-values. *P*-values > 0.1 were denoted as non-significant (n.s.), *p*-Values between 0.1 and 0.05 are shown in italics to indicate tendencies, and *p*-Values ≤ 0.05 indicating significance were shown in regular font. The number of observations per treatment is indicated by the number in brackets.

The BE application showed significant improvements in aboveground nutrient content and nutrient use efficiency. The response of aboveground P content (40%) was slightly larger than the improvement of aboveground N content (28%) (Figures 8A, 9A). Crop types differed in their response to BE application regarding aboveground N and P content. Aboveground N content was especially enhanced in legumes (81%) (Figure 8B). Also, cereals and oilseed crops have the tendency to attain positive outcomes with an increase of 37%. For aboveground P content, differences were marginal among cereals, legumes, and fruits (61% to 87%) (Figure 9B). The application time after sowing led to the highest increase with regard to aboveground N and P content (64% and 73%) (Figures 8C, 9C). Both aboveground nutrient content responses did not differ in the test systems (Figures 8D, 9D). Soil (79%), foliar (49%), and a combination of different methods of application (90%) improved aboveground P content significantly (Figure 9E). In the case of aboveground N content, only soil application (104%) led to significant increases while seed application (29%) showed a tendency to increase N content (Figure 8E). Aboveground N content was significantly enhanced in strongly acidic environments by BE application (116%) (Figure 8F). For aboveground P content, a tendency toward higher efficiencies in lower pH levels was indicated; however, aboveground P content was again lower in strongly acidic soils (27% increase) compared to slightly acidic soils (72% increase) (Figure 9F). For both nutrients, the highest improvements of aboveground content were achieved in lower-middle-income economies with 71% increases for N and 76% increases for P (Figures 8G, 9G). In accordance with the increase of biomass, extracts improved aboveground P content by 110% when examining it with the extended model (Figures 10A,B). In the case of aboveground P content, extracts and a dual combination treatment achieved significant positive results and AMF and others enhanced aboveground P content in tendency, whereas N was significantly increased by dual combinations and in tendency improved by N-fixing bacteria.

**FIGURE 11:**
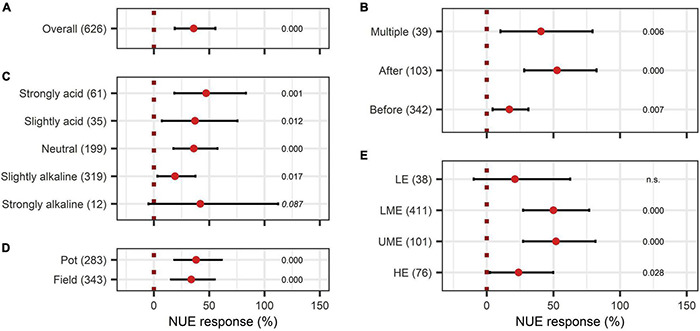
Response of Nitrogen use efficiency (NUE). **(A)** Overall NUE increase of all observations (median), **(B–E)** are subgroup analyses on NUE: **(B)** time of application relative to sowing, **(C)** pH categories, **(D)** test system, **(E)** country grouping (LE: low-income economies, LME: lower-middle-income economies, UME: upper-middle-income economies, HE: high-income economies). Dots mark the median, lines mark the 95% confidence interval. If the line crosses the dotted line, the impact of bioeffectors is not significant (α = 0.05). Numbers beside are the *P*-values. *P*-values > 0.1 were denoted as non-significant (n.s.), *p*-Values between 0.1 and 0.05 are shown in italics to indicate tendencies, and *p*-Values ≤ 0.05 indicating significance are shown in regular font. The number of observations per treatment is indicated by the number in brackets.

**FIGURE 12:**
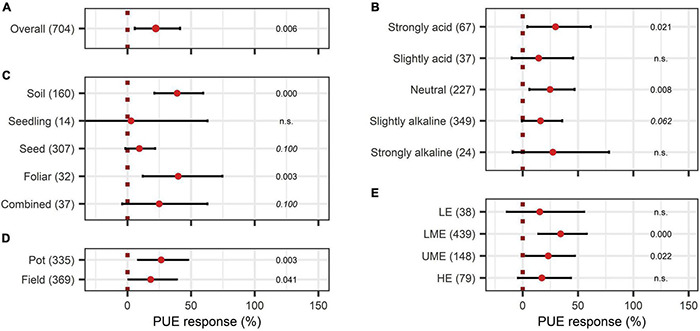
Response of Phosphate use efficiency (PUE). **(A)** Overall PUE increase of all observations (median), **(B–E)** are subgroup analyses on PUE: **(B)** pH levels, **(C)** mode of application, **(D)** test system, **(E)** country grouping (LE: low-income economies, LME: lower-middle-income economies, UME: upper-middle income economies, HE: high-income economies). Dots mark the median and lines mark the 95% confidence interval. If the line crosses the dotted line, the impact of bioeffectors is not significant (α = 0.05). Numbers beside are the *p*-Values. *p*-Values > 0.1 were denoted as non-significant (n.s.), *p*-Values between 0.1 and 0.05 were shown in italics to indicate tendencies, and *p*-Values ≤ 0.05 indicating significance were shown in regular font. The number of observations per treatment is indicated by the number in brackets.

The overall effect of N-use efficiency was significantly enhanced by BE application with an increase of 36% (Figure 11A). Also, P-use efficiency was significantly improved by 22% (Figure 12A). To achieve enhanced nutrient-use efficiency, the application time needs to be considered for N, whereas the mode of application was the decisive factor for P. To increase N-use efficiency the most, an application time point after sowing should be preferred, which resulted in the highest improvements of 53% compared to 41% for multiple treatments and 17% for an application time before or at sowing (Figure 11B). For P-use efficiency, foliar application (40%) as well as soil application (39%) were the most suitable methods (Figure 12C). Moreover, N-use efficiency and P-use efficiency were dependent on soil pH. In both cases, the trend of higher efficiencies in lower pH levels was approximately visible (Figures 11C, 12B). While the test system (field or pot) did not alter the results for N-use efficiency (Figure 11D), pot trials (26%) showed a higher median than field trials (18%) for P-use efficiency (Figure 12D). In the models of N- and P-use efficiency, country grouping was included as a factor. The median response of BE application on N- and P-use efficiency was especially high in lower-middle-income (50% and 34%, respectively) and upper-middle-income countries (52% and 23%, respectively), whereas it was slightly reduced in low- (21% and 15%, respectively) and high-income economies (24% and 17%, respectively) (Figures 11E, 12E).

**FIGURE 13:**
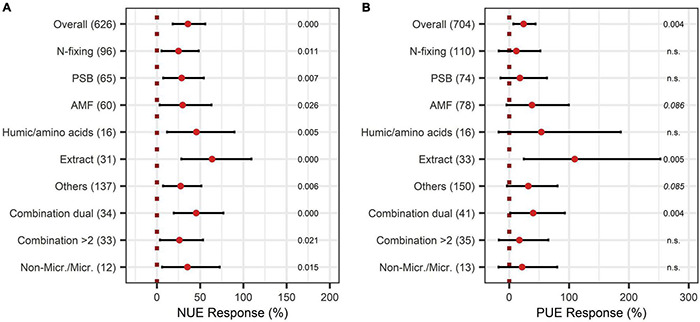
Response of Nitrogen use efficiency (NUE) **(A)** and Phosphate use efficiency (PUE) **(B)** by different BE types according to the expanded model. Dots mark the median, lines mark the 95% confidence interval. If the line crosses the dotted line, the impact of bioeffectors is not significant (α = 0.05). Numbers beside are the *p*-Values. *p*-Values > 0.1 were denoted as non-significant (n.s.), *p*-Values between 0.1 and 0.05 were shown in italics to indicate tendencies, and *p*-Values ≤ 0.05 indicating significance were shown in regular font. The number of observations per treatment is indicated by the number in brackets.

When the models of N-use efficiency were expanded by BE types, N-use efficiency was shown to be significantly improved by all BE types (Figure 13A). In particular, extracts and also humic and amino acids, and the dual combination treatment attained high responses for both nutrients, with 64%, 46%, and 45% for N-use efficiency (Figure 13A) and 109%, 54%, and 40% for P-use efficiency, respectively (Figure 13B). For the application of microbes, a combination of two different inoculants appeared to be more beneficial to improve N- and P-use efficiency than the application of only one BE type (Figures 13A,B).

## Discussion

### The General Impact of Bioeffectors

In this metaanalysis, BE application had a positive impact on the shoot and root biomass, yield, and analyzed quality indicators as well as on aboveground nutrient uptake.

Since the outcome of a metaanalysis strongly depends on the input data, it can be assumed that the current results could be slightly biased due to a higher chance of publication success for studies with positive BE effects and missing unpublished study data. But given the number of studies published on the positive impact of BEs, it is likely that the results indicate an existing effect but may overestimate the magnitude. The higher biomass translated directly into higher yields underlining the economic opportunity going along with BE application.

The benefit of BEs on nutrient uptake and nutrient use efficiency was already summarized by previous reviews (Kumar and Aloke, 2020; Rouphael and Colla, 2020a,b). In the current study, the strong increase of plant macronutrient uptake by BE application, in general, could be confirmed. Moreover, BEs can contribute to the production of high-quality food and fodder. In this study, they enhanced protein content and soluble solids content, which are some of the most important indicators for taste and nutritional value. These results agree with findings from previous studies, which investigated other quality indicators (Paradikovic et al., 2011; Maach et al., 2021).

The comparison of different BE types according to the categorization above was not included in any model for the analysis of plant growth or quality traits. One could hypothesize that the type of BE is not crucial for the success of the usage, which is consistent with recent findings from the metaanalysis by Lekfeldt et al. (2021). However, BEs are selected for studies based on the experimental setting and research question, and only a few studies directly compared BE types. Thus, the results of this analysis could be biased due to non-random selection of the BE types. The expansion of the selected models with BE types resulted in only slight increases of the AIC value. Significant deviations from the main effect of extracts and humic/amino acids on shoot biomass, as well as the effect of a combination of microbial and non-microbial products on shoot biomass and yield, could be detected with the expanded model. Additionally, a combination of two different microbial strains could exploit synergies and increased biomass as well as aboveground N content, which was also highlighted by a recent review (Santoyo et al., 2021). The increase in aboveground P content and P-use efficiency by PSB was not outstanding. Raymond et al. (2020) suggested that P solubilizing capabilities of bacteria suffice to cover the demands of the bacteria, but not to support plant P acquisition. Therefore, the improvement of aboveground P content might be caused by an improved spatial acquisition rather than an enhanced mobilization of bound phosphate in soil. Nevertheless, the P in the microbial biomass pool enters the nutrient cycle and becomes available to plants in the long term. The improvement of P nutrition through extracts, humic, and amino acids, however, did not go along with a proportional root growth increase. Thus, it cannot be related exclusively to enhanced spatial P acquisition. Other mechanisms and interactions related to the application of humic and amino acids and extracts need to be considered, such as humic–metal complexes, influence on soil biota, and increase of nutrient translocation (Anitha, 2020; Shukla and Prithiviraj, 2021). However, these mechanisms need to be clarified in future experimental studies.

In accordance with previous metaanalyses (Rubin et al., 2017; Schmidt and Gaudin, 2018), stronger positive effects on shoot biomass in pot trials than in field trials could be confirmed. This could also explain higher overall responses of root biomass compared to shoot biomass as root biomass was only investigated in pot experiments. Weinmann and Neumann (2020) suggested that BEs efficiency is higher in pot trials due to the limited soil volume, which leads to an artificially high concentration of the BE inoculant around the densely growing root. In contrast to pot trials, the root is not restricted in its growth direction in the field. This could hinder the establishment of symbiosis with BEs, as they are outcompeted by native microbial communities, which are spatially closer to the root (Weinmann and Neumann, 2020). However, in the results of aboveground N and P content, the higher efficiency of BEs in pot trials could not be identified. To clarify this contradiction, it would be helpful to reveal underlying mechanisms to avoid results that are influenced by experimental approaches (e.g., higher application rates in pot trials, controlled conditions in the greenhouse) rather than by the test system.

### Crop Types and Soil pH Determined the Effect Size of Bioeffectors

It was shown that the potential in achieving certain inoculation aims depends on the crop type as yield, aboveground nutrient contents, as well as quality traits response differed with the crop type. This may be the result of specialized symbiosis of plants and microbes to acquire nutrients in the soil, such as N-fixing bacteria and legumes, while other crop types lack this intense mutualistic interaction. In this study, the increase of aboveground N content in cereals and legumes indicates the development of more effective symbiosis with soil microorganisms, which is known to exist in these crop types (Rosenblueth et al., 2018). However, the potential amount of nitrogen fixation is lower in cereals than in legumes (Cooper and Scherer, 2012), which is also reflected by the results of this study.

Soil physicochemical characteristics can impact the success of BE application (Weinmann and Neumann, 2020). In this study, it was highlighted that the pH level of the soil strongly influences the outcome of a BE application. As mentioned above, most of the BEs can mobilize insoluble nutrients, such as P, in several ways. BEs act as a counterpart against nutrient fixation in soil by acidification, which is regarded as the most crucial impact of soil microbes (Raymond et al., 2020). However, the results imply that this mechanism is restricted to slight pH decreases as the efficacy of BEs decreased in almost any case in strongly alkaline soils. Additionally, the results from this analysis suggest that the highest benefit of BE application occurs in strongly acidic environments with the highest increase in shoot and root biomass. It also shows that the effectiveness of BE application to improve plant growth traits tends to increase with decreasing soil pH, especially for shoot biomass. In these soils, nutrients, such as P, become insoluble due to reaction with Al and Fe (Margenot et al., 2018). By an enhanced root growth, as demonstrated in this study, the acquisition of scarce soluble nutrients can be improved. BEs could support root growth in acidic environments by chelating detrimental Fe and Al-cations and forming metal–humic complexes (Khan et al., 2009; Anitha, 2020).

### The Right Time and Right Way to Use Bioeffectors

It remains an open research question whether better-adapted application methods could improve the effectiveness of BE application. Application methods should be highly effective and easily adoptable in common agricultural schemes (Weinmann and Neumann, 2020). In the present metaanalysis, it was shown that investigated methods of application had a significant impact on growth parameters, quality traits, and aboveground nutrient content. Foliar and soil application seem to be the most promising application methods to achieve reliable growth gains, improve nutritional quality, and increase nutrient uptake. The application time influenced aboveground N and P content and N-use efficiency. Consistently, an application time after sowing obtained highest efficiencies in all cases, in which application time was included in the model.

The country grouping provided a broad indicator for agronomical production regimes. Most of the investigated effects showed a dependency on management; however, in almost none of the cases the impact was negative, and for yield, country grouping was not included in the model. It could therefore be argued that the benefits from BE application are realizable in any region. Yet, differences in efficiency across countries could be detected regarding shoot biomass, aboveground N and P content, as well as N- and P-use efficiency. This may be due to different baselines of N- and P-use efficiency in the regions and therefore, varying improvement opportunities for growth and nutrient uptake. In the study of Omara et al. (2019), low-income economies and high-income economies obtained N-use efficiencies above the global average, while China and India, which contributed to a great extent to the data for this study, showed N-use efficiencies below the global average (Omara et al., 2019). One could hypothesize that BEs can especially support these countries with a low nutrient use efficiency, while in countries that already have a high efficacy, BEs cannot contribute much to ameliorate nutrient use efficiency even more.

## Conclusion and Outlook

Reducing mineral fertilizer input while maintaining high yield levels is crucially important in sustainable agricultural production. As demonstrated in this study, BEs can contribute to achieve this goal by improving plant growth by 26%, yield by 30%, and P and N uptake by 40% and 28%, respectively. In contrast to studies focusing on single microbial BEs, we could show that non-microbial BEs and composite products with combinations of different microbial and non-microbial BEs are most promising as they increased biomass by 40%–60%. However, the results are not consistent. The application time, the application mode (e.g., foliar, placement, and combinations), and soil characteristics, especially the pH level, need to be considered to guarantee effective action of BEs. In summary, the effects of BEs on crop growth traits were strongest for the following: application after sowing; foliar application, a combination of several different application methods, and a soil application; as well as under acidic soil conditions especially regarding biomass response and nutrient uptake. Harmonizing BE types, application mode, crop type, and soil characteristics should be the focus of future studies. Further research is needed to clarify the mechanisms of non-microbial products, especially their influence on plant P uptake.

## Data Availability Statement

The data analyzed in this study is subject to the following licenses/restrictions: The datasets, SAS scripts and R scripts of the current study are available from the corresponding author on reasonable request. Requests to access these datasets should be directed to MH, m.herrmann@uni-hohenheim.de.

## Author Contributions

HY and MH conceptualized the study. MH and YW collected the data. JH and MH implemented the statistical analysis. MH drafted and wrote the manuscript. HY and TM complemented the work. HY, TM, and XC supervised the work. All authors read and revised the manuscript and approved the final version.

## Conflict of Interest

The authors declare that the research was conducted in the absence of any commercial or financial relationships that could be construed as a potential conflict of interest.

## Publisher’s Note

All claims expressed in this article are solely those of the authors and do not necessarily represent those of their affiliated organizations, or those of the publisher, the editors and the reviewers. Any product that may be evaluated in this article, or claim that may be made by its manufacturer, is not guaranteed or endorsed by the publisher.
