# Supplementary material for: A Global Network Meta-Analysis of the Promotion of Crop Growth, Yield, and Quality by Bioeffectors
Source: Front Plant Sci. 2022 Mar 1;13:816438. doi: 10.3389/fpls.2022.816438 (PMC8921507; doi:10.3389/fpls.2022.816438)
Supplement: Supplementary file 1 [file Table_1.DOCX]

Supplementary Material

Table 1: Conversion factors for estimation of biomass in kg/ha, Values are to convert g/plant in kg/ha, for trees and vine to convert kg/tree in kg/ha.

| *Wheat* | 3000 (*KWS* 2021) | *Lentil* | 1200 *(GRDC* 2018) | *French Bean* | 333.33 (*Reddy* 2018) | *Pepper* | 42 *(Brodie* n.y.) |
| --- | --- | --- | --- | --- | --- | --- | --- |
| *Rice* | 2000 *(Kahlown* et al. *2001)* | *Tomato* | 22 *(Wikifarmer* n.y.b) | *Cotton* | *9*0 *(Wikifarmer* n.y.c) | *Sour orange* | 250 (*Wikifarmer* n.y.d) |
| *Broccoli* | 29 *(Wikifarmer* n.y. a) | *Maize* | 65 *(IPNI* 2013) | *Soybean* | 200 *(Grain Sa* 2015) | *Miscanthus* | 20 *(Pyter* et al. n.y.*)* |
| *Sorghum* | 200 *(Seedcogroup* n.y.) | *Sesame* | 167 *(Nadeem* et al. 2015) | *Chickpea* | 300 *(Patil* et al 2021) |  |  |
| *Pomegranate* | 400 *(Anonymous* n.y.) | *Mint* | 200 *(AgriFarming* n.y.) | *Bean* | 250 *(Starke Ayres* n.y.) |  |  |

**References:**

*Agrifarming* (n.y.). Mint Farming. Available via: <https://www.agrifarming.in/mint-farming> Accessed 18. August 2021.

*Anonymous* (n.y.): Pomegranate. Available via: <http://nhb.gov.in/report_files/pomegranate/POMEGRANATE.htm> Accessed 18. August 2021.

*Grain Sa* (2015): Recommendations for soybean plant establishment. Available via: [https://www.grainsa.co.za/recommendations-for-soybean-plant-establishment Accessed 18. August 2021](https://www.grainsa.co.za/recommendations-for-soybean-plant-establishment%20Accessed%2018.%20August%202021).

*GRDC* (2018): GrowNote Lentil. Available via: <https://www.grdc.com.au/__data/assets/pdf_file/0026/366182/GrowNote-Lentil-South-4-Planting.pdf> Accessed 18. August 2021.

*IPNI* (2013): Plant population and spacing for maize. Available via: <http://seap.ipni.net/article/SEAP-3031> Accessed 18. August 2021.

*Kahlowan, M. A., Raoof, A., Hanif, M.* (2001): Plant population effect on paddy yield. *J.Drainage Water Manage.* 5(1): 1-5.

*KWS* (2021): Aussaat Weizen. Available via [*https://www.kws.com/de/de/beratung/aussaat/weizen/*](https://www.kws.com/de/de/beratung/aussaat/weizen/) Accessed 18. August 2021.

*Nadeem, A., Kashani, S., Ahmed, N., Buriro, M., Saeed, Z., Mohammad, F., Ahmed, S.* (2015): Growth and Yield of Sesame (Sesamum indicum L.) under the Influence of Planting Geometry and Irrigation Regimes. *American J. Plant Sci.* 6:980-986.

*Patil, S. B., Mansur, C. P., Gaur, P. M., Salakinkop, S. R., Alagundagi, S. C.* (2021): Planting Density Affected Dry Matter Production, Partitioning, and Yield in Machine Harvestable Chickpea Genotypes in the Irrigated Ecosystem. Int. *J. Plant Prod* 15:29-43.

*Pyter, R., Voigt, T., Heaton, E., Dohleman, F., Long, S.* (n.y.): Growing Giant Miscanthus in Illinois. Available via: http://www.miscanthus.illinois.edu/wp-content/uploads/growersguide.pdf Accessed 18. August 2021.

*Reddy* (2018): French Beans Cultivation. Available via: <https://www.asiafarming.com/french-beans-cultivation> Accessed 18. August 2021.

*Seedcogroup* (n.y.): Sorghum Growers Guide. Available via: <https://www.seedcogroup.com/sites/default/files/Sorghum%20Growers%20Guide.pdf> Accessed 18. August 2021.

*Starke Ayres* (2019): Bean Production Guidelines. Available via: <https://www.starkeayres.com/uploads/files/Bean-Production-Guideline-2019.pdf> Accessed 18. August 2021.

*Wikifarmer* (n.y.)a: Wie man Brokkoli gewinnbringend anbaut. Available via : <https://wikifarmer.com/de/wie-man-brokkoli-gewinnbringend-anbaut-brokkoli-kommerzanbau/> Accessed 18. August 2021.

*Wikifarmer* (n.y.)b: Commercial tomato farming. Available via: <https://wikifarmer.com/commercial-tomato-farming/> Accessed 18. August 2021.

*Wikifarmer* (n.y.)c: Cotton sowing seeding rate and plant population. Available via: <https://wikifarmer.com/cotton-sowing-seeding-rate-and-plant-population/> Accessed 18. August 2021.

*Wikifarmer* (n.y.)d: Orange tree harvest and yields. Available via: <https://wikifarmer.com/orange-tree-harvest-and-yields/> Accessed 18. August 2021.

Table 2: Akaike Information Criterion (AIC) and overall mean for the selected model and the with BE type expanded model.

|  | AIC | AIC with BE | Mean | Mean with BE |
| --- | --- | --- | --- | --- |
| Shoot Biomass | 926.87 | 938.06 | 0.2249 | 0.2420 |
| Yield | 2314.3 | 2334 | 0.2602 | 0.2860 |
| Root Biomass | 557.29 | 560.15 | 0.5240 | 0.4952 |
| Aboveground N content | 1202.8 | 1212.8 | 0.2496 | 0.2718 |
| Aboveground P content | 1133 | 1140.1 | 0.3357 | 0.3041 |
| Nitrogen use efficiency | 565.51 | 581.92 | 0.3073 | 0.3060 |
| Phosphate use efficiency | 639.51 | 656.36 | 0.2010 | 0.2163 |

Table 3: Results of the model selection. Included parameters after model selection are indicated by an x in the respective column.

|  | Bioeffector types | Pot vs. Field | Application Time | Mode of application | pH | Crop type | Perennial vs. Annual | Country grouping |
| --- | --- | --- | --- | --- | --- | --- | --- | --- |
| Shoot Biomass |  | x |  | x | x |  |  | x |
| Yield |  | x |  |  |  | x |  |  |
| Root Biomass |  |  |  | x | x |  |  |  |
| Soluble Solids |  | x |  |  | x | x |  |  |
| Protein |  | x |  | x | x | x | x | x |
| Aboveground N content |  | x | x | x | x | x |  | x |
| Aboveground P content |  | x | x | x | x | x |  | x |
| N-Use efficiency |  | x | x |  | x |  |  | x |
| P-Use efficiency |  | x |  | x | x |  |  | x |

**Table 4:** Summary of the significant influencing factors for each model. Per parameter vector (in each row) the parameters are listed in descending order of their influence.

|  | Shoot Biomass | Yield | Root Biomass | Soluble solids | Protein | N content | P content | NUE | PUE |
| --- | --- | --- | --- | --- | --- | --- | --- | --- | --- |
| BE type | Extract  Non-Microbes and Microbes mixed  Humic/amino acids  Dual combination | Non-Microbes and Microbes mixed  All others | AMF  Non-Microbes and Microbes mixed  Dual combination |  |  | Dual combination | Extract  Dual combination | Extract  All others | Extract  Dual combination |
| Pot/Field | Pot | Pot  Field |  | Field | Field | Pot | Field  Pot | Pot  Field | Pot  Field |
| Crop type |  | Herb  Fruit  Legume  Vegetable  Cereal |  | Legume | Legume | Legume | Fruit  Cereal  Legume |  |  |
| Perennial or annual |  |  |  |  | Perennial |  |  |  |  |
| pH | <5.5  5.5-6.5 |  | <5.5  5.5-6.5  7.5-8.5 | 7.5-8.5 | 6.5-7.5  5.5-6.5 | <5.5  6.5-7.5 | 5.5-6.5  6.5-7.5 | <5.5  <8.5 | <5.5  6.5-7.5 |
| Application mode | Combined  Soil  Foliar |  | Combined  Seed  Soil  Foliar |  | Foliar  Soil | Soil | Combined  Soil  Foliar |  | Foliar  Soil |
| Application time |  |  |  |  |  | After | After | After  Multiple  Before |  |
| Geographic region | Lower middle income  Upper middle income |  |  |  | Upper middle income | Lower middle income | Lower middle income  Upper middle income | Upper middle income  Lower middle income  High income | Lower middle income  Upper middle income |
